# Cost-effectiveness analysis of depression case finding followed by alerting patients and their GPs among older adults in northern England: results from a regression discontinuity study

**DOI:** 10.1192/bjo.2025.782

**Published:** 2025-06-26

**Authors:** Qian Zhao, David John Torgerson, Kerry Jane Bell, Joy Ann Adamson, Caroline Marie Fairhurst, Sarah Cockayne, Jennie Lister, Kalpita Baird, David Ekers

**Affiliations:** Department of Health Sciences, University of York, UK; Tees Esk and Wear Valleys NHS Foundation Trust, York, UK

**Keywords:** Depression, screening, older adults, cost-effectiveness, quality-adjusted life-years

## Abstract

**Background:**

In the UK, around 1 in 4 adults over 65 years suffers from depression. Depression case finding followed by alerting patients and their general practioners (GPs) (screening + GP) is a promising strategy to facilitate depression management, but its cost-effectiveness remains unclear.

**Aims:**

To investigate the cost-effectiveness of screening + GP compared with standard of care (SoC) in northern England.

**Method:**

Conducted alongside the CASCADE study, 1020 adults aged 65+ years were recruited. Participants with baseline Geriatric Depression Scale (GDS) ≥5 were allocated to the intervention arm and those >5 to SoC. Resource use and EQ-5D-5L data were collected at baseline and 6 months. Incremental cost-effectiveness ratio was calculated. Non-parametric bootstrapping was performed to capture sampling uncertainty. The results are presented using cost-effectiveness acceptability curves. Sensitivity analyses were conducted to assess the robustness of primary findings. Subgroup analyses were undertaken to examine the cost-effectiveness among participants with more comparable baseline characteristics across treatment groups.

**Results:**

Screening + GP incurred £37 more costs and 0.006 fewer quality-adjusted life years than SoC; the probability of the former being cost-effective was <5% at a £30 000 cost-effectiveness threshold. Sensitivity analyses confirmed the base-case findings. Subgroup analyses indicated that screening + GP was cost-effective when patients with baseline GDS 2–7, 3–6 and 4–5, respectively, were analysed.

**Conclusions:**

Screening + GP was dominated by SoC in northern England. However, subgroup analyses suggested it could be cost-effective if patients with more balanced baseline characteristics were analysed. Economic evaluations alongside randomised controlled trials are warranted to validate these findings.

Depression is characterised by a long-lasting feeling of sadness and loss of pleasure or interest in daily activities.^1^ It ranks among the most debilitating mental disorders and is a major contributor to the global health burden.^2^ According to the World Health Organization (WHO), approximately 3.8% of the global population suffers from depression; prevalence escalates to 5.7% among those aged 60 years and older.^3^ Recent studies revealed that older people with depression incurred direct medical costs 1.7-fold higher than those without depression, and higher levels of depression are significantly associated with deteriorated future quality of life.^4,5^

In the UK, approximately 1 in 4 adults over the age of 65 years is experiencing depression; 85% of individuals with depression do not receive any support from the National Health Service (NHS), and 90% of those affected do not consult any health specialist.^6,7^ The problem is attributable to the difficulties faced by general practitioners (GPs) and other primary healthcare workers in identifying depressive patients. These challenges arise because older adults often present with somatic rather than psychological symptoms, unlike younger adults.^7,8^ Furthermore, the diagnosis of depression among the older population is complicated by the high prevalence of comorbidities, including cancer, neurological disorders, cardiovascular diseases and arthritis.^9^

Based on an evidence review from 2020, depression screening using validated tools to systematically identify individuals at risk of depression is currently not recommended by the UK National Screening Committee (NSC) due to suboptimal diagnosis accuracy, uncertain benefits and unclear status of how depression is identified and managed.^10^ The evidentiary basis for older adults is even more scarce and less clear, with no studies in the UK investigating the clinical effectiveness and cost-effectiveness of screening.^11–14^

## Aims

The aim of this cost-effectiveness analysis (CEA) was to assess the cost-effectiveness of screening followed by alerting patients and their GP (screening + GP) compared with standard of care (SoC). This study reports the methods and results of the economic evaluation embedded in the CASCADE (Case finding for depression in primary care: a regression discontinuity design) study, following the Consolidated Health Economic Evaluation Reporting Standards (CHEERS) 2022.^15^

## Method

### Study design and participants

CASCADE is a two-arm, pragmatic, multicentre study using a regression discontinuity design targeting older adults (≥65 years) not currently experiencing depression or receiving treatment for it. The study strictly complied with the ethical standards of national and institutional committees on human research and the Helsinki Declaration. Ethical approval was granted by the Yorkshire and The Humber–Leeds West Research Ethics Committee (reference no. 22/YH/0119).^16^

CASCADE was conducted across 15 GP practices in the North of England, spanning NHS regions including the North-East and North Cumbria, Greater Manchester (including Manchester, Salford and Stockport) and Yorkshire and Humber (including Leeds, Sheffield and Bradford). These practices were identified through Clinical Research Networks (CRN) recommendations, established collaborations and as direct outreach to managers of Research and Development and Clinical Commissioning Groups. Between 18 November 2022 and 30 June 2023, 9184 recruitment packs were mailed out by 15 GP practices, from which 846 (9.2%) eligible patients with written consent were enrolled. Between 9 June and 14 July 2023 a second wave of recruitment was conducted among 7 of the 15 GP practices which had patients that had not been contacted, to enhance recruitment efficiency. In total, 6665 invitation texts were sent and 174 participants (2.6%) further enrolled, bringing total enrollment to 1020 participants. A detailed summary on the geographic and demographic details of recruitment areas is provided in Supplementary Materials, Appendix A, available at https://doi.org/10.1192/bjo.2025.782.

The regression discontinuity design is a quasi-experimental method considered one of the most robust alternatives to randomised controlled trials (RCTs) in estimating treatment effects.^17,18^ It minimises selection bias by leveraging the quantitative assignment variable (QAV) and its inherent measurement error. In this design, individuals are assigned to treatment or control group based on whether their baseline QAV score falls above or below a predefined cut-off. Due to natural measurement error, some participants may be misclassified by chance, ensuring that those near the threshold are statistically similar in both observed and unobserved characteristics. This mimics the balance achieved through randomisation, strengthening causal inference.^18^

In CASCADE, Geriatric Depression Scale-15 (GDS-15) was chosen as the QAV, an instrument that has been extensively validated and used for assessing depression in older adults. Scores range from 0–4 (normal), 5–8 (mild depression), 9–11 (moderate depression) to 12–15 (severe depression).^19^ Due to ethical considerations and recommendations from GPs and Patient and Public Involvement members, the cut-off point of QAV was set at 5, indicating mild depression. Participants and their GP were notified about the score by the research team if their baseline GDS was ≥5. All participants were followed up on their GDS at 6 months and then investigated to determine whether there was discontinuity (or ‘jump’) at the cut-off point in the regression line between baseline and 6-month GDS.^20^

Baseline demographics and resource use were obtained from these participants. Six months following baseline, GDS and EQ-5D-5L scores were collected to determine whether the intervention had helped improve depression, and resource use was also recorded to facilitate the CEA.

### Intervention and comparator

As per the nature of regression discontinuity study, for those scoring GDS ≥5, the following took place: (a) the York Trials Unit (YTU) informed participants of their GDS scores via a postal letter and that their score indicated at least mild depression; (b) the YTU informed the participant’s GP of their GDS score using a secure file transfer system; (c) the GP made a clinical decision on the next course of action (screening + GP).

Regarding those with baseline GDS <5, no action was taken, despite the fact that all participants were sent signposting information to mental health services and were still able to access usual care from their GP and other healthcare services if needed, reflecting the current standard of care (SoC).

### Health-related outcome measure

The primary health-related outcome of the CEA was quality-adjusted life year (QALY), which was calculated by combining the health-related quality of life (HrQoL), in the form of utility, and the period for which the utility is assumed to apply. The utility was derived based on participants’ responses to the EQ-5D-5L instrument. This instrument comprises five domains (mobility, self-care, usual activities, pain or discomfort, and anxiety or depression), each of which is measured with five levels (no problems, slight problems, moderate problems, severe problems and extreme problems).^21^ Responses to the EQ-5D-5L instrument were mapped to EQ-5D-3L based on participants’ age band and gender, to derive the EQ-5D-3L utility for QALY computation.^22,23^ QALYs of each participant were generated with the area under the curve (AUC) approach.^24^ Due to the 6-month time horizon, discounting of QALYs was not applied.

### Measurement of resource use

Healthcare resource utilisations were measured via baseline and 6-month patient-reported questionnaires. In line with National Institute for Health and Care Excellence recommendations, the UK NHS and Personal Social Services (PSS) perspective was adopted for costing in the base case.^22^ All mental health-related resource utilisations funded by NHS and social services were included: (a) community care: consultations with GP/nurse/other healthcare practitioners; (b) hospital care: Accident and Emergency or Urgent Care Centre visits and overnight in-patient stays; (c) NHS mental health services (measured only at 6 months): consultations with psychologist, psychiatrist, community psychiatric nurse, etc.; (d) medications for depression (measured only at 6 months): citalopram, dapoxetine, escitalopram, fluoxetine, etc.; and (e) social care: social worker visit and paid home care worker visit.

Societal perspective was also adopted in sensitivity analysis, where travel costs, productivity losses, help from charities, private mental healthcare services and self-care activities were further considered.

### Valuation of resource use

The total costs were derived by attaching the unit cost to the number of units of each resource utilisation consumed by participants. The unit costs were mainly sourced from national databases, including the 2021/2022 National Cost Collection for the NHS and Unit Costs of Health and Social Care reports by the Personal Social Services Research Unit (PSSRU), supplemented by published literature.^25–27^ Productivity loss was valued based on the median weekly earnings from the UK Office for National Statistics.^28^ The unit costs for medications were obtained from the NHS Prescription Cost Analysis database for England.^29^

Specifically, when dosage information was not available from the participants, it was replaced with the daily dose recommended by the British National Formulary; when frequencies (number of days taking the medication) were not available, these were replaced with the average value of the available observations in the data-set.^30^ Regarding the unit cost of charity support, assigning accurate costs to the assistance participants received (e.g. toenail cutting, help with using laptops, wet room showers, etc.) was challenging. Therefore, we used the hourly rate of a paid home care worker as a proxy, because these services were predominantly domestic in nature. A summary of unit costs of resource utilisations is available in Supplementary Materials, Appendix B.

All costs are expressed in 2021–2022 Great British pounds (GBP). When appropriate, unit costs were inflated to 2021–2022 prices with the NHS Cost Inflation Index.^31^ Due to the 6-month time horizon, discounting of costs was not applied.

### Missing data

Missing data at baseline and 6 months were imputed according to the recommendations by Faria et al.^32^ Missing pattern of baseline and 6-month costs by category and utility were plotted to determine whether missing data of particular categories could be imputed aggregately. Logistic regression was performed to investigate the association between missingness of total costs/QALYs and baseline covariates, as well as the association between 6-month utility/costs and previous observations at baseline. The above diagnosis indicated that certain categories of cost could be imputed as a whole to ensure a stable imputation model without losing too much information from the available data, and the most likely missing mechanism is missing at random (MAR). A more detailed description of missing data is available in Supplementary Materials, Appendices C and D.

Under MAR, missing costs and QALYs at each assessment point were imputed by treatment group using multiple imputation with chained equations (MICE), where predictive mean matching (PMM) drawn from the five nearest neighbours (*k*_nn_ = 5) was conducted, ensuring the robustness of imputed values to violations of the normality assumption. Predictive covariates included baseline age, gender, ethnicity, living arrangement, education, GDS score, comorbidities and utility. Prior to inclusion in the imputation model, missing baseline covariates were imputed with simple imputation methods, in which mean imputation was used to impute continuous variables and mode imputation for categorical variables.^32^

As a rule of thumb, 40 imputations were performed because the highest percentage of missing data in all variables was 36%.^33^ Furthermore, because it is usually impossible to investigate whether data are missing not at random (MNAR), MNAR assumption was tested in sensitivity analysis using pattern mixture modelling.^34^

### Statistical and economic analysis

The primary outcome of the CEA was incremental cost-effectiveness ratio (ICER), which was compared against the conventional threshold (£20 000–30 000 per QALY) used in the UK. To account for the correlation between costs and QALYs, the imbalances of baseline characteristics between treatment groups, and sampling uncertainty, seemingly unrelated regression equations (SURE) controlling for baseline age, gender, ethnicity, living arrangement, education, comorbidities, costs and utility, were bootstrapped 1000 times. The bootstrapped pairs of incremental costs and QALYs were plotted on the cost-effectiveness plane (CE-plane), and the probability of screening + GP being cost-effective under a range of cost-effectiveness thresholds is presented using the cost-effectiveness acceptability curve (CEAC).

All analyses were conducted using Stata (version 18 for Windows), based on an intention-to-treat approach.

### Sensitivity analyses

Sensitivity analyses were performed to test the robustness of the cost-effectiveness findings. First, a CEA based on complete cases was conducted, in which only those participants with full observations of costs and QALYs at both baseline and 6 months were included. The ratio of SoC versus screening + GP among complete cases was not manually adjusted to match that of the base case, to avoid potential selection bias. Second, a CEA from societal perspective was conducted. Third, a CEA was performed where missing data were imputed under MNAR assumption using pattern mixture modelling. A scale parameter (set at 10%) was used to modify the imputed utility and cost data from the imputation data-sets under MAR assumption.^34^ A total of 7 MNAR scenarios were tested based on different combinations of scale parameters, where we assumed that, compared with the observed values, those with missing data are associated with: (a) 10% HrQoL reduction in both arms; (b) 10% cost increase in both arms; (c) 10% HrQoL and 10% cost increase in both arms; (d) 10% HrQoL reduction in screening + GP arm; (e) 10% HrQoL reduction in SoC arm; (f) 10% cost increase in screeing + GP arm; and (g) 10% cost increase in SoC arm.

### Subgroup analyses

The validity of the regression discontinuity design relies on the similarity of patients ‘just below’ and ‘just above’ the threshold, which approximates randomisation.^20^ However, this approach may result in large disparities of baseline characteristics among treatment groups, particularly between participants with GDS scores well below 5 and those significantly above 5, which might lead to biased cost-effectiveness estimates. Therefore, to explore the cost-effectiveness of the intervention based on more comparable participants, we performed subgroup analyses by restricting participants to those with baseline GDS of 0–9, 1–8, 2–7, 3–6 and 4–5, respectively, in the cost-effectiveness analyses.

## Results

### Participants

In total, 1020 participants were included in the base-case CEA, where 863 were allocated to SoC and 157 to the screening + GP group. Among those, 392 had full observations of QALYs and costs data at baseline and 6-month time points, constituting the complete cases. The baseline characteristics by treatment group of both populations are presented in Table 1. Both complete-case and base-case populations predominantly consisted of participants who identified as White.

**Table 1:** Baseline characteristics by treatment group

Baseline characteristics	Base case (*n* = 1020)		Complete case (*n* = 392)	
	SoC (*n* = 863)	SG (*n* = 157)	SoC (*n* = 356)	SG (*n* = 36)
Male, *n* (%)	452 (52.4)	87 (55.4)	221 (62.1)	22 (61.1)
Ethnicity, *n* (%)				
White	854 (99.0)	156 (99.4)	353 (99.2)	36 (100.0)
Mixed/multiple	1 (0.1)	1 (0.6)	1 (0.28)	0 (0)
Asian/Asian British	3 (0.4)	0 (0)	0 (0)	0 (0)
Black/Black British, Caribbean or African	5 (0.6)	0 (0)	2 (0.56)	0 (0)
Living alone, *n* (%)	202 (23.4)	56 (35.7)	85 (23.9)	16 (44.4)
Qualification, *n* (%)				
No formal qualifications	126 (14.6)	37 (23.6)	33 (9.3)	7 (19.4)
O-Level, CSE, School Certificate, GCSE	182 (21.1)	26 (16.6)	91 (25.6)	6 (16.7)
A-Level, Higher School Certificate, AS	60 (7.0)	15 (9.6)	29 (8.2)	4 (11.1)
NVQ, qualification related to clerical	169 (19.6)	33 (21.0)	76 (21.4)	7 (19.4)
Degree or higher	326 (37.8)	46 (29.3)	127 (35.7)	12 (33.3)
Comorbidity, *n* (%)				
Arthritis	302 (35.0)	72 (45.9)	116 (32.6)	14 (38.9)
Cancer	57 (6.6)	9 (5.7)	25 (7.0)	1 (2.8)
Cardiovascular condition	275 (31.9)	56 (35.7)	123 (34.6)	16 (44.4)
Chronic pain	88 (10.2)	50 (31.9)	35 (9.8)	8 (22.2)
Diabetes	80 (9.3)	21 (13.4)	30 (8.4)	6 (16.7)
Neurological disorder	10 (1.2)	5 (3.2)	5 (1.4)	3 (8.3)
Osteoporosis	50 (5.8)	20 (12.7)	13 (3.7)	7 (19.4)
Respiratory condition	109 (12.6)	28 (17.8)	44 (12.4)	6 (16.7)
Stroke	16 (1.9)	9 (5.7)	6 (1.7)	3 (8.3)
Mean (s.d.) age	73.9 (6.1)	73.5 (6.6)	73.7 (5.9)	73.1 (7.0)
Mean (s.d.) GDS score	1.3 (1.3)	7.6 (2.7)	1.1 (1.2)	7.2 (2.9)
Mean (s.d.) utility	0.83 (0.15)	0.59 (0.25)	0.84 (0.14)	0.64 (0.26)

AS, Advanced Subsidiary; CSE, Certificate of Secondary Education; GCSE, General Certificate of Secondary Education; GDS, Geriatric Depression Scale; NVQ, National Vocational Qualification; SG, screening + alerting participants and general practitioner (GP); SoC, standard of care.

Among the base-case population, the percentage of male participants (52.4 *v*. 55.4%), proportion of White participants (99.0 *v*. 99.4%) and mean age (73.9 *v*. 73.5 years) were similar across treatment groups. Compared with SoC, participants in the screening + GP group were less educated, were associated with a higher incidence of comorbidities, worse GDS score and lower utility scores and were more likely to live alone, as expected by the cohort definition (GDS ≥5). The disparity between treatment groups highlights the uniqueness of the regression discontinuity design, distinguishing it from RCTs.

On the other hand, patients in the complete-case population displayed better baseline characteristics (lower GDS score and higher utility) than the base-case population; this is because patients who were able to complete the questionnaires are likely to be healthier. Among complete cases, only 36 out of 157 (22%) screening + GP participants provided complete data, compared with 356 out of 863 (41%) SoC participants. This difference is attributed to the composition of the SoC group, which primarily included participants with normal or less-than-mild depression and overall better health, making them more likely to complete the questions than those in the screening + GP group. Additionally, approximately 60% of participants in both arms of the complete-case population were male, compared with around 50% in the base-case population. This difference may be explained by logistic regression findings, which showed female participants having significantly higher odds of missing total cost and utility data compared with males.

### Utility and QALYs

Table 2 shows the average EQ-5D-3L utility by treatment group when missing utility scores were not imputed (complete case), and when they were imputed (base case). At baseline and 6-month follow-up, the absolute utility scores of the SoC group were consistently higher than the screening + GP group in both the base case and complete case, which could be attributed to the regression discontinuity design, where patients with lower baseline GDS and higher utility were allocated to the SoC group. Comparing the utility changes between 6 months and baseline, however, patients in the screening + GP group underwent around 0.03 increment while the SoC group was subject to slight decrement. This highlights the potential benefit of the screening + GP strategy. After calculating total QALYs of the base-case population using the AUC approach (without baseline covariate adjustment), it was found that screening + GP produced 0.113 fewer QALYs than SoC during the 6-month follow-up. However, caution is required when interpreting the unadjusted comparison, considering that the screening + GP group were associated with worse baseline utility.

**Table 2:** Average EQ-5D-3L utility and total QALYs by treatment group

Utility	Base case, mean (95% CI)		Complete case, mean (95% CI)	
	SoC (*n* = 863)	SG (*n* = 157)	SoC (*n* = 356)	SG (*n* = 36)
Baseline	0.831 (0.830, 0.833)	0.586 (0.579, 0.592)	0.842 (0.827, 0.857)	0.638 (0.540, 0.716)
6-month	0.825 (0.823, 0.827)	0.616 (0.610, 0.622)	0.839 (0.825, 0.853)	0.669 (0.587, 0.738)
Total QALYs	0.414 (0.413, 0.415)	0.301 (0.298, 0.303)	0.420 (0.414, 0.427)	0.327 (0.283, 0.364)

QALY, quality-adjusted life year; SoC, standard of care; SG, screening + alerting participants and general practitioner (GP).

### Resource use and costs

As shown in Table 3, from an NHS and PSS perspective the average costs associated with the screening + GP group were £233.70 over 6 months, but only £26.10 for the SoC group, in the base-case population. Costs across all subcategories for the screening + GP group were notably higher, with hospital-based care being the leading cost driver. This aligns with the fact that the screening + GP group comprised less healthy individuals who would incur greater healthcare utilisations, such as Accident and Emergency or Urgent Care Centre visits and overnight in-patient stays. Restricting the participants to complete case only, there was a reduction in the between-group cost difference compared with base case.

**Table 3:** Average costs of resource use by treatment group over 6 months

Costs	Base case, mean (95% CI)		Complete case, mean (95% CI)	
	SoC (*n* = 863)	SG (*n* = 157)	SoC (*n* = 356)	SG (*n* = 36)
NHS and PSS	26.1 (19.9, 31.6)	233.7 (189.2, 281.0)	5.0 (1.6, 11.1)	57.1 (18.0, 141.5)
Community-based care	0.9 (0.8, 1.0)	33.1 (31.5, 34.8)	0.8 (0.3, 2.3)	17.3 (8.0, 33.3)
Hospital-based care	19.2 (13.5, 24.3)	169.8 (117.9, 214.9)	1.2 (0, 7.0)	0.0 (0, 0)
Medications	0.0 (0, 0)	3.5 (2.9, 4.3)	0.0 (0, 0)	1.8 (0, 3.6)
NHS mental health services	2.0 (1.7, 2.3)	10.2 (9.0, 11.4)	2.1 (0.1, 7.2)	10.7 (0, 30.8)
PSS-based services^a^	4.0 (3.5, 4.6)	17.0 (14.9, 19.4)	0.9 (0, 2.7)	27.3 (0, 101.7)
Private perspective	70.5 (68.0, 73.2)	111.7 (104.7, 119.6)	52.7 (39.4, 70.4)	134.7 (37.9, 287.0)
Travel costs	4.3 (4.1, 4.5)	11.8 (10.5, 13.0)	3.8 (2.1, 6.2)	2.0 (0.4, 4.5)
Other costs^b^	66.3 (63.7, 68.9)	99.9 (93.0, 107.4)	49.0 (36.0, 66.7)	132.7 (37.0, 283.5)
Societal perspective	96.6 (90.0, 103.2)	345.4 (298.4, 393.6)	57.8 (43.3, 75.5)	191.8 (66.1, 423.6)

NHS, National Health Service; PSS, personal social services; SoC, standard of care; SG, screening + alerting participants and general practitioner (GP).

a PSS-based costs include cost of social worker and paid home worker visit.

b Other costs include the cost of charity, productivity loss of carers and patients, private mental health care services and self-care activities.

When broader costs were considered from a societal perspective, the total cost difference between groups further increased in both base-case and complete-case populations, because patients with more severe depressive conditions require additional care from society, their friends and family members.

It is worth noting that the cost difference could have also be attributed to outliers (cases with exceptionally high costs). For instance, one outlier in the screening + GP group incurred a cost of £21 767.54 for hospital-based care, equivalent to 38 nights of in-patient stay. Notwithstanding the potential bias due to outliers, these were not excluded from the analysis because of their real-world occurrence and the possibility of such events. For a detailed summary on resource use over 6 months, please refer to Supplementary Materials, Appendix E.

### Base-case results of CEA

**Fig. 1:**
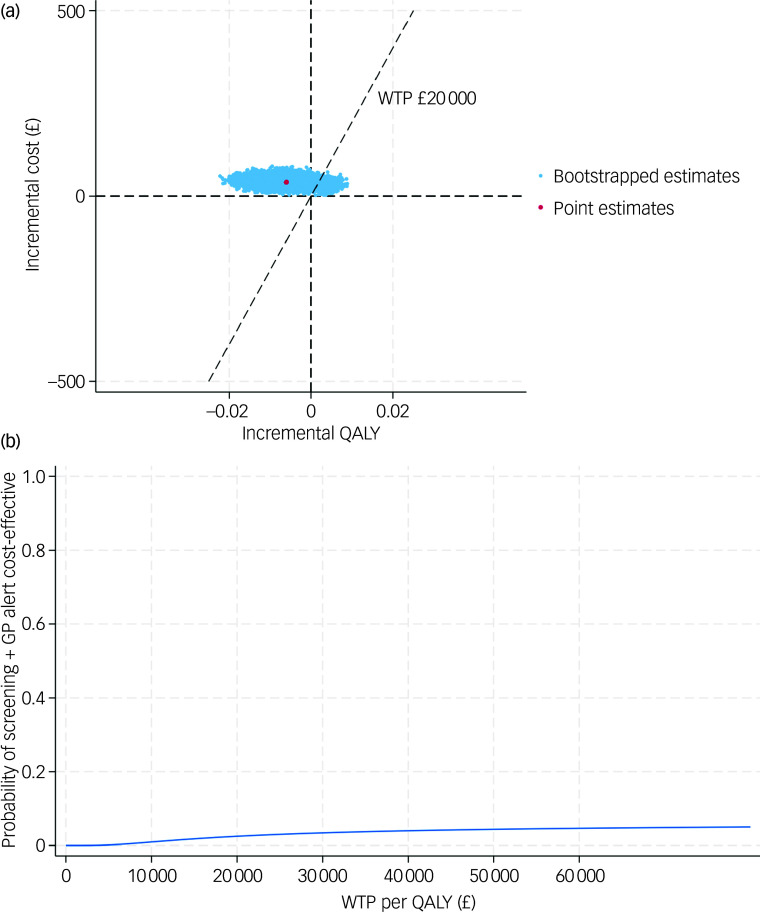
(a) cost-effectiveness plane (base case); (b) cost-effectiveness acceptability curve (base case). QALY, quality-adjusted life year; WTP, willingness to pay; GP, general practitioner.

After further accounting for sampling uncertainty and imbalance of baseline characteristics, patients in the screening + GP group incurred higher costs (£37, 95% CI £21–56) and slightly lower QALYs (−0.006, 95% CI −0.014 to 0.002) over a 6-month follow-up, which is equivalent to approximately 2.19 fewer days with perfect health. The CE-plane in Fig. 1(a) illustrates the 1000 cost–QALY replicated pairs from non-parametric bootstrapping. As shown, the bootstrapped pairs predominantly clustered to the left side of the *y* axis and upper aspect of the *x* axis, indicating that screening + GP was highly likely to be dominated (higher costs but fewer benefits) by SoC. The CEAC in Fig. 1(b) further confirms this finding, where the probability of the screening + GP group being cost-effective was only 2.5% given a £20 000 threshold.

### Sensitivity analyses

In order to test the robustness of the base-case results, a set of sensitivity analyses were conducted. The complete case-based CEA indicated that the screening + GP group incurred £28 more costs and 0.006 fewer QALYs than SoC, while the CEA adopting societal perspective yielded similar results (£34 greater costs and 0.006 fewer QALYs). Under MNAR assumption, the seven tested scenarios all showed that the screening + GP group cost more but generated less health benefit. The findings of the above sensitivity analyses were in line with the base case, confirming that screening + GP is a dominated strategy.

**Fig. 2:**
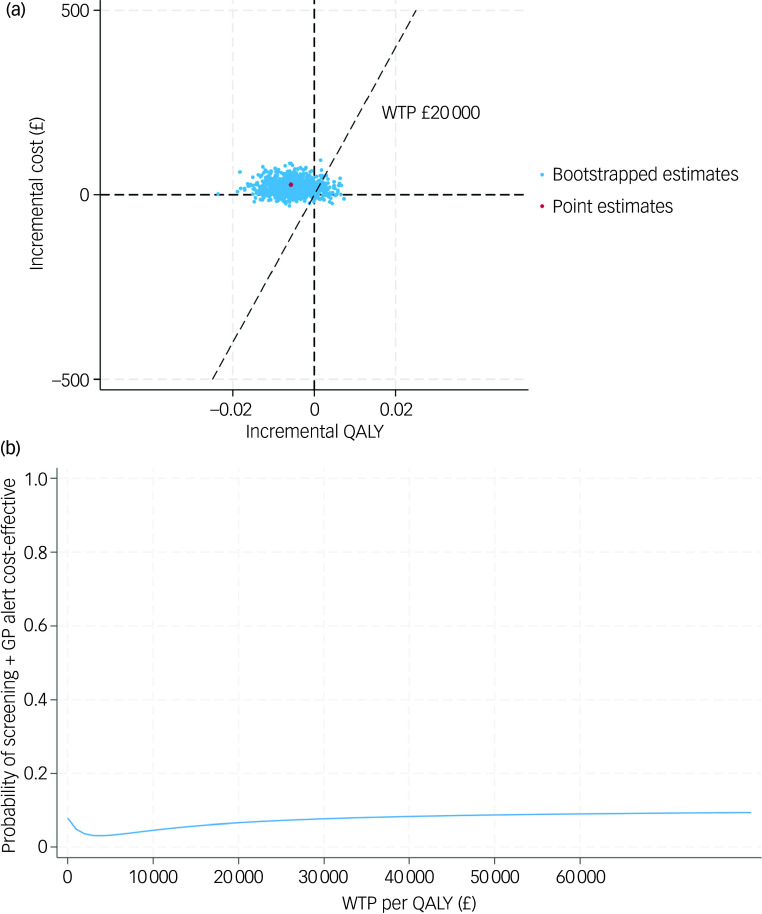
(a) cost-effectiveness plane (complete case); (b) cost-effectiveness acceptability curve (complete case). QALY, quality-adjusted life year; WTP, willingness to pay; GP, general practitioner.

**Fig. 3:**
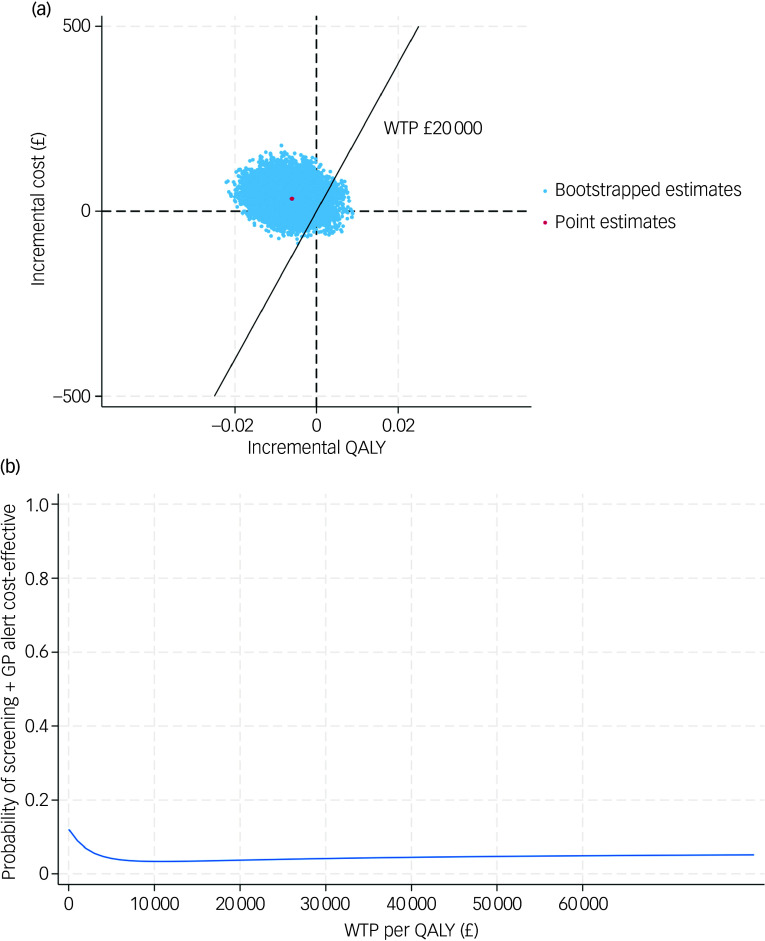
(a) cost-effectiveness plane (societal perspective); (b) cost-effectiveness acceptability curve (societal perspective). QALY, quality-adjusted life year; WTP, willingness to pay; GP, general practitioner.

**Fig. 4:**
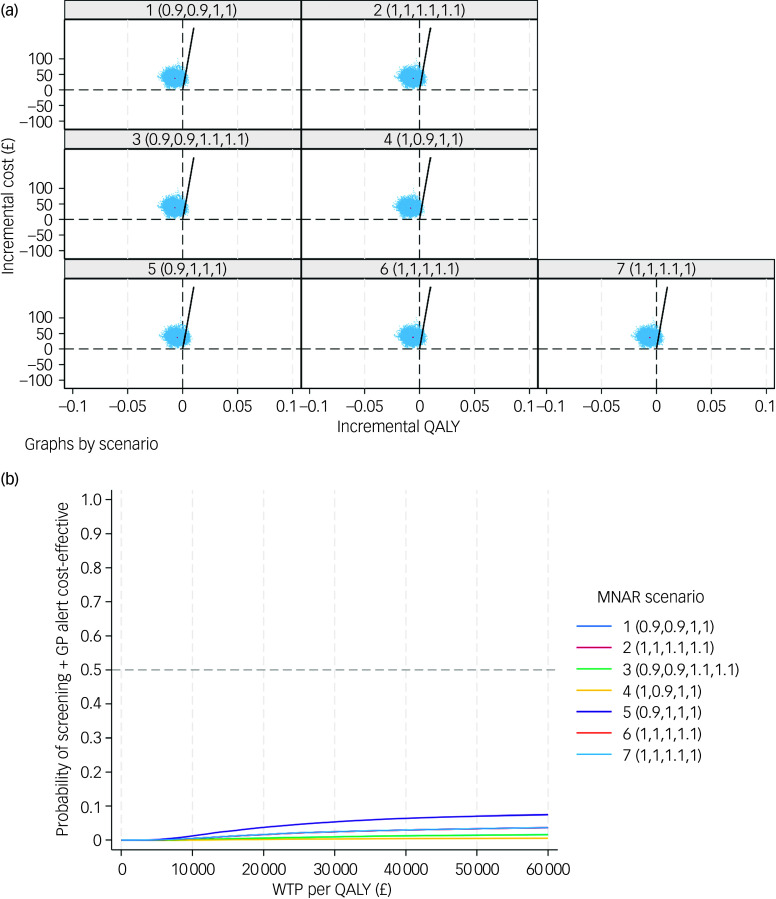
(a) cost-effectiveness planes under seven MNAR scenarios; (b) cost-effectiveness acceptability curves under seven MNAR scenarios. MNAR, missing not at random; QALY, quality-adjusted life year; WTP, willingness to pay; GP, general practitioner.

The CE-planes and CEACs of complete-case analysis, societal perspective-based analysis and CEA under seven MNAR scenarios are presented in Figs 2, 3 and 4.

### Subgroup analyses

In subgroup analyses, we step-wisely restricted participants to those with baseline GDS of 0–9, 1–8, 2–7, 3–6 and 4–5 in the CEA to gradually approximate the RCT design. In order to further confirm whether the subgroup of patients share sufficiently similar baseline characteristics that could be considered as an RCT, the baseline characteristics of participants with baseline GDS 4–5, as well as 3–6, are presented in Table 4, where a chi-squared test was performed for categorical variables and two-sample *t*-test for continuous variables. According to the table, most of the characteristics being compared were balanced, meaning that the participants in both treatment groups in subgroup analyses were more comparable than base case. Although there were still statistically significant between-group differences in terms of EQ-5D-3L utility and distribution of qualifications, those exceptions could happen just by chance due to the limited sample sizes of subgroups.

**Table 4:** Comparison of baseline characteristics between participants who scored slightly below and above the GDS cut-off point

Baseline characteristics	Baseline GDS					
	4 (*n* = 66)	5 (*n* = 44)	*P*-value^a^	3–4 (*n* = 174)	5–6 (*n* = 75)	*P*-value
Male (%)	37.9	54.6	0.09	48.3	58.7	0.13
Ethnicity (%)						
White	100	97.7	0.22	100	98.7	0.13
Mixed/multiple	0	2.3		0	1.3	
Asian/Asian British	0	0		0	0	
Black/Black British, Caribbean or African	0	0		0	0	
Living alone (%)	25.8	43.2	0.06	25.3	37.3	0.05
Qualification (%)						
No formal qualifications	12.1	22.7	0.01	14.4	22.7	0.02
O-Level, CSE, School Certificate, GCSE	19.7	9.1		21.3	9.3	
A-Level, Higher School Certificate, AS	1.5	9.1		5.2	10.7	
NVQ, qualification related to clerical	12.1	29.6		15.5	24.0	
Degree or higher	54.6	29.6		43.7	33.3	
Comorbidity (%)						
Arthritis	50	47.7	0.82	48.3	44.0	0.54
Cancer	6.1	9.1	0.55	8.1	6.7	0.71
Cardiovascular condition	36.4	38.6	0.81	36.2	34.7	0.82
Chronic pain	18.2	29.6	0.16	17.8	28.0	0.07
Diabetes	16.7	18.2	0.84	12.1	14.7	0.57
Neurological disorder	1.5	2.3	0.77	1.7	2.7	0.63
Osteoporosis	7.6	9.1	0.78	6.3	10.7	0.24
Respiratory condition	10.6	6.8	0.50	11.5	10.7	0.85
Stroke	4.6	4.6	1.00	2.3	4.0	0.46
Mean (s.d.) age	74.9 (7.4)	74.0 (6.1)	0.53	75.0 (7.2)	73.5 (6.1)	0.13
Mean (s.d.) utility	0.72 (0.19)	0.62 (0.21)	0.01	0.75 (0.15)	0.65 (0.20)	0

AS, Advanced Subsidiary; CSE, Certificate of Secondary Education; GCSE, General Certificate of Secondary Education; GDS, Geriatric Depression Scale; NVQ, National Vocational Qualification; SoC, standard of care; SG, screening + alerting participants and general practitioner (GP).

a Chi-squared test was conducted for categorical variables, and *t*-test for continuous variables.

**Fig. 5:**
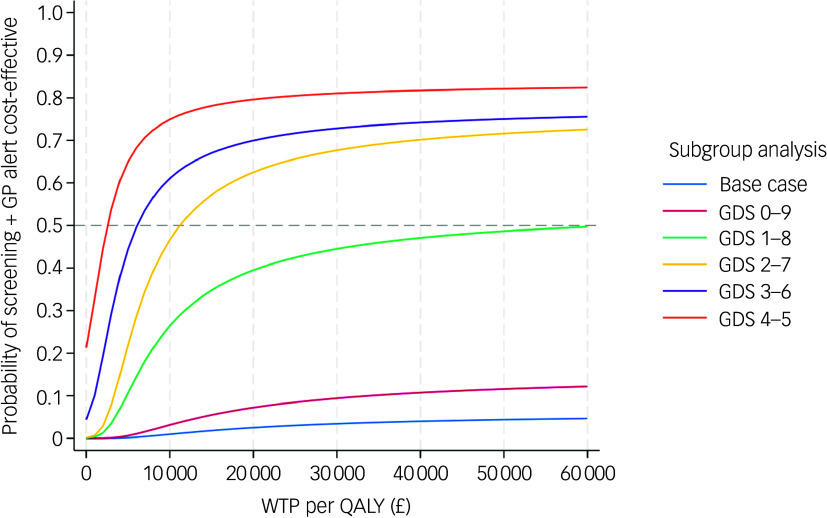
Pooled cost-effectiveness acceptability curves of subgroup analyses and base-case analysis (NHS and PSS perspective, imputed, controlled for baseline covariates). GDS, Geriatric Depression Scale; NHS, National Health Service; PSS, personal social services; QALY, quality-adjusted life year; WTP, willingness to pay; GP, general practitioner.

The subgroup analyses revealed that the cost-effectiveness of the screening + GP group exhibited an improving trend as the baseline characteristics of both arms became more comparable. The ICERs were –£9488 (dominated), £63 630, £11 218, £6098 and £2551 for subgroups with baseline GDS scores of 0–9, 1–8, 2–7, 3–6 and 4–5, respectively. The pooled CEACs of the base case and five subgroups shown in Fig. 5 clearly demonstrate improvement in the probability of the screening + GP group being cost-effective in subgroup analyses compared with the base case. The results indicate that, when the baseline characteristics were more balanced (GDS 4–5, 3–6 and 2–7), the screening + GP group could represent a highly cost-effective strategy.

## Discussion

### Principal findings

Given the scarcity of RCTs investigating the effectiveness and cost-effectiveness of depression screening among older adults in the UK, our economic evaluation embedded within an regression discontinuity study has generated valuable economic evidence. The base-case CEA found that alerting patients and their GP following identification of mild or above-depression symptoms incurred higher costs but lower QALYs compared with standard of care from a NHS and PSS perspective. Sensitivity analyses consistently produced similar cost-effectiveness results, indicating that these are robust to analytical perspective and how missing data were handled. Those findings support current recommendations by the UK NSC.^10^ However, the subgroup analyses, limited to population with more comparable baseline GDS (GDS 4 *v*. 5, GDS 3–4 *v*. 5–6 and GDS 2–4 *v*. 5–7), demonstrated that screening + GP is cost-effective.

Without considering sampling uncertainty and baseline covariate imbalance, the screening + GP group was associated with 0.113 fewer QALYs and £207.60 higher costs than the screening-only group. However, QALY difference diminished to 0.006 and cost difference dropped to £37 after uncertainty was handled with bootstrapping and covariates were adjusted with a regression-based approach.^24,35^ The decrease in QALY and cost difference compared with unadjusted results highlights the impact of baseline imbalance on the cost-effectiveness results due to the nature of the regression discontinuity design. However, we believe that the conventional regression-based approach for adjusting baseline covariates is insufficient to produce near bias-free cost-effectiveness estimates based on the regression discontinuity study. This is due to the more pronounced baseline imbalances inherent in regression discontinuity designs compared with those observed in RCTs.^35^ Therefore, we further performed subgroup analyses, which have demonstrated that screening + GP is a cost-effective strategy among certain subgroups.

### Strengths and limitations

To the best of our knowledge, this is the first attempt to undertake an economic evaluation embedded in a prospective regression discontinuity study. It is also the first study to evaluate the cost-effectiveness of alerting older patients and their GPs following a diagnosis of depression in the primary care setting within the UK. This study was conducted by strictly following the manual of health technology evaluations issued by NICE, and is reported following the CHEERS checklist.^15,22^ It was based on a broad spectrum of health resource use (from both NHS and PSS and a societal perspective) and quality of life data prospectively collected from participants in 15 GP practices in the North of England, enabling us to capture the real-world costs and health implications of depression screening among older adults in northern England. We extensively tested the robustness of cost-effectiveness findings to various assumptions. When dealing with missing data, we carried out diagnosis on the mechanism of missingness to inform the most robust imputation method, and additionally tested seven potential MNAR scenarios to reassure the robustness of base-case findings. Subgroup analyses were also undertaken to address the issue of baseline imbalances.

However, our study also had several limitations. First and foremost, this is an RD study rather than an RCT. Participants with mild, moderate or severe depression were assigned to the intervention group while those with normal or less-than-mild depression were assigned to the usual care group. This prevents us from conducting subgroup analyses stratified by depression severity to explore the treatment effects and cost-effectiveness within these sub-populations, among which treatment outcomes and management strategies differ substantially. Moreover, despite the methodology for conducting economic evaluation alongside RCT being well developed, there is no established guidance on how to perform economic evaluation embedded in regression discontinuity study. Due to the lack of RCTs and the urgent need for cost-effectiveness evidence in this area, we borrowed the recommended methods from trial-based economic evaluations in the base-case analysis and carried out extensive subgroup analyses, aiming to approximate a trial-based economic evaluation to reveal the ‘true’ cost-effectiveness estimates, although the small sample size of the subgroups may have affected the reliability of the findings.^24,35,36^ While our approach cannot fully eliminate baseline imbalances, it represents a good practice in the absence of formal guidance.

Second, more than half of the data-set was missing, with only 38.4% of participants providing complete observations of QALYs and costs (from a societal perspective) at baseline and 6 months. Such substantial missing data could potentially lead to biased estimates and affect the reliability of the conclusions drawn. However, in the base-case analysis, which considered the perspectives of the NHS and PSS, the proportion of participants providing full costs and QALY data increased to 61.1%. This considerable improvement in data completeness helps mitigate the uncertainties caused by data missingness. It is also important to note that the high rate of missing data was anticipated and inevitable, because the study participants are predominantly older adults with comorbidities and depressive symptoms who may not have been sufficiently well to comprehend and complete the questionnaires.

Third, the distribution of particular types of cost data (e.g. cost of NHS mental health services) exhibited a two-part pattern, where there was a predominant proportion of zero cases; this suggests that a two-part model might have been a better choice. However, using that method would neglect the correlation between cost and effects. Additionally, after fitting two-part models using both simple linear regression and a generalised linear regression model as the second part of the model, we found that the estimated incremental costs were £23 and 35, respectively, fluctuating by only a small magnitude from our base case (£37). This means that using SURE rather than the two-part model does not alter the cost-effectiveness results, and further confirmed the validity of using the SURE approach. Further in-depth comparison on the optimal way to model costs data is beyond the scope of this study, but is highly encouraged in future research.

Fourth, several unit costs could not be identified from either PSSRU, National Cost Collection or published literature. For instance, participants reported various types of charity support, such as toenail cutting, for which costs were difficult to quantify. To address this, we applied the hourly rate of a paid home care worker as a proxy. While this may not perfectly reflect the actual costs, the relatively low unit costs and infrequency of such services (reported by only 6 out of 1020 participants) minimise its impact. Since both treatment groups were handled consistently, the incremental estimates and cost-effectiveness results are unlikely to be biased.

Fifth, response rates for both recruitment packs and invitation texts were <10%, which limited study sample size. This may be attributed to several factors, including stigma surrounding mental health, lack of awareness of symptoms of depression but seeing low mood as a routine part of growing older, and ‘research fatigue’, with patients asked to participate in numerous studies in research active practices.

Last, the CASCADE study was undertaken among 15 GP practices solely located in the North of England, where the prevalence of undiagnosed depression and socioeconomic disparities is relatively higher than in the rest of the country.^37,38^ Additionally, a White population constitutes >95% of the study population, although this was due solely to the demographic composition of the research area rather than to an intentional exclusion of participants from non-White ethnic groups. Given these factors, the findings may not be generalisable to other contexts, especially for regions with more optimal healthcare access, mental health support and diverse demographics.

### Future research

Due to the constraints of resource and time, our study was restricted to the short-term cost-effectiveness of intervention. Long-term modelling, although not within the research scope, is warranted to explore lifetime cost-effectiveness. In light of the growing influence of real-world data (RWD) on health technology assessments, it might play a valuable role in informing long-term modelling. We strongly recommend that researchers engage several UK-based RWD databases, such as Clinical Practice Research Datalink (CPRD), Hospital Episode Statistics (HES) and Mental Health Services Dataset (MHSDS), to explore the long-term cost-effectiveness of depression case finding. More importantly, when more robust clinical evidence from RCT becomes available, an economic evaluation alongside RCT is necessary.

Depression among older adults is a global challenge. To generalise our findings, we encourage researchers to conduct economic evaluations in other healthcare settings or among different populations, such as younger individuals, those with severe depression or comorbidities and more ethnically diverse populations. When doing so, it is important to adjust for differences such as the analytical perspective, clinical pathways, types and unit costs of resource use and value sets for estimating health benefits accordingly.

Given that healthcare practitioners involved in our study expressed concerns that participants with severe depression may be less likely to complete the GDS questionnaire, coupled with the overall high level of missing data, future research should focus on developing and validating questionnaires that achieve higher response rates to facilitate data collection. Specifically, we recommend the use of shorter questionnaires with clear and accessible language, as well as replacing self-reported data with investigator-assisted interviews and investigator-completed forms in future studies. These strategies may help improve data completeness, accuracy and participant adherence among populations with severe mental health and other similar conditions.

Last, while our study primarily assessed the cost-effectiveness of screening, because preventive strategies (public mental health awareness campaigns or artificial intelligence-enhanced chatbots) and the provision of convenient access to follow-up treatments following the diagnosis of depression may play a crucial role in reducing depression incidence and improving the quality of life of depressed patients, future research is also warranted to investigate the associated clinical and economic benefits.

In conclusion, the CEA embedded in the CASCADE study showed that alerting patients and their GP following the diagnosis of depression is not cost-effective compared with standard of care in a primary care setting in northern England. Such a conclusion remains consistent in sensitivity analyses. The CEA based on subgroups with more similar baseline characteristics (GDS 4–5, 3–6 and 2–7) indicated that it is highly cost-effective.

## Supporting information

Zhao et al. supplementary material Zhao et al. supplementary material

## Data Availability

The data-set that supported the findings of this study is not publicly available due to its containing information that could compromise the privacy of research participants. However, the Stata code for data analysis can be shared upon reasonable request from the corresponding author, Q.Z.

## Supplementary material

The supplementary material is available online at https://doi.org/10.1192/bjo.2025.782
