# Supplementary material for: Cost-effectiveness analysis of depression case finding followed by alerting patients and their GPs among older adults in northern England: results from a regression discontinuity study
Source: BJPsych Open. 2025 Jun 26;11(4):e125. doi: 10.1192/bjo.2025.782 (PMC12247059; doi:10.1192/bjo.2025.782)
Supplement: Zhao et al. supplementary material [file S2056472425007823sup001.docx]

**Appendix A. Geographic and demographic details of recruitment areas**

| **Geographical coverage** | **Number of GP practices** | **Type of GP practices** | **Number of recruitment packages sent** | | **Number of recruited participants** | | **Ethnicity distribution** |
| --- | --- | --- | --- | --- | --- | --- | --- |
|  |  |  | **Wave 1** | **Wave 2** | **Wave 1** | **Wave 2** |  |
| Northeast and North Cumbria | 2 | Rural | 276 | 450 | 17 | 12 | 100% White |
| Greater Manchester | 2 | Urban | 1150 | 0 | 67 | 0 | 95.5% White, 1.5% Other (Mixed/multiple, Asian/Asian British or Black/Black British), 3.0% missing |
| Yorkshire and Humber | 11 | 7 urban GP practices  1 coastal GP practice  3 rural GP practices | 7758 | 6215 | 762 | 162 | 98.6% White, 1% Other (Mixed/multiple, Asian/Asian British or Black/Black British), 0.4% missing |
| Note: Wave 1 = recruitment via postal recruitment packs; Wave 2 = text recruitment | | | | | | | |

**Appendix B. Unit costs of health resource use and associated data sources**

| **Cost category** | **Unit cost^1^ (£)** | **Unit** | **Reference** |
| --- | --- | --- | --- |
| **Mental health-related community care** | | | |
| GP consultations | 47.98 | session | Assuming one hour per session  PSSRU 2017, page 185, table 12.1  Average hourly cost of a team member of the NHS community mental health team (CMHT) for older people with mental health problems^2^ |
| Nurse consultations | 47.98 | session |  |
| Consultations with other health care practitioners | 47.98 | session |  |
| **Mental health-related hospital care** | | | |
| A&E or an Urgent Care Centre | 82.90 | visit | National cost collection 2021/2022 (T01NA and T04NA); Weighted average of cost of A&E and urgent care centre, based on the unit cost and number of attendances per year |
| Inpatient overnight stay | 572.83 | stay | National cost collection 2021/2022; Non-Elective Inpatient - Short Stay; WD09Z |
| **Social care** | | | |
| Social worker visit | 52.00 | visit | PSSRU 2021, page 122, table 11.1, assuming one hour per visit |
| Home visits from paid home worker | 33.00 | visit | PSSRU 2021, page 126, table 11.5, assuming one hour per visit; Unit cost was derived by taking the average cost of visit provided for private purchases and social services |
| **NHS mental health services** | | | |
| Psychologist consultation for depression | 360.98 | session | National cost collection 2021/2022; Service code 656, "Clinical Psychology Service" |
| Psychiatrist consultation for depression | 493.35 | session | National cost collection 2021/2022; Service code 715, "OLD AGE PSYCHIATRY SERVICE", consultant-led |
| Community psychiatric nurse consultation for depression | 565.32 | session | National cost collection 2021/2022; Service code 715, "OLD AGE PSYCHIATRY SERVICE", non-consultant-led |
| Group therapy for depression | 228.00 | session | National cost collection 2021/2022; A01AG, Other Therapist, Adult, Group |
| Outreach worker consultation for depression | 25.00 | session | PSSRU 2021; page 128, table 11.7, assuming one hour per session |
| IAPT service for depression | 132.00 | session | PSSRU 2021; page 34, table 2.1 |
| Counsellor consultation for depression | 51.00 | session | PSSRU 2021; page 80 table 6.10; used child as a proxy, assuming one-hour session |
| Private counselling session | 51.00 |  | Assume the same as counsellor |
| First contact mental health consultant | 360.98 |  | Assume the same as psychologist |
| **Help from charities** | 33.00 | visit | Assume the cost of charity worker is the same as hourly rate of paid home worker to simplify |
| **Medications** | | | |
| Citalopram (Cipramil) 20 mg | 0.09 | mg | PCA  BNF code: 0403030D0AAAAAA |
| Dapoxetine (Priligy) 30 mg | 1.10 | mg | PCA  BNF code: 0704060A0BBACAA |
| Escitalopram (Cipralex) | 0.13 | mg | PCA  BNF code: 0403030X0AAAAAA |
| Fluoxetine (Prozac or Oxactin) | 0.05 | mg | PCA  BNF code: 0403030E0AAAAAA |
| Fluvoxamine (Faverin) | 0.32 | mg | PCA  BNF code: 0403030L0AAAAAA |
| Paroxetine (Seroxat) | 0.08 | mg | PCA  BNF code: 0403030P0AAAAAA |
| Sertraline (Lustral) | 0.02 | mg | PCA  BNF code: 0403030Q0AAAAAA |
| Vortioxetine (Brintellix) | 4.91 | mg | PCA  BNF code: 0403040ABBBAAAA |
| **Productivity loss** | | | |
| Median wages | 114.98 | day | ONS 2023; Assuming 5 working days per week; £574.9 per week (all employees, including full-time and part-time) |
| **Private mental health services (according to patient-reported categories of services)** | | | |
| Private counsellor | 55 | session | How Much Does Private Therapy Cost? -Select Psychology *https://selectpsychology.co.uk/blog/mental-health/how-much-does-private-therapy-cost* |
| Psychotherapist | 80 | session | How Much Does Private Therapy Cost? -Select Psychology *https://selectpsychology.co.uk/blog/mental-health/how-much-does-private-therapy-cost/* |
| Abbreviations: A&E: accident and emergency; BNF: British National Formulary; GP: general practitioner; IAPT: Improving Access to Psychological Therapies; NHS: National Health Service; PCA: Prescription Cost Analysis; PSSRU: Personal Social Services Research Unit  Note: ^1^All costs presented in the table have been inflated with appropriate Hospital & Community Health Services (HCHS) indices based on the original pricing year. Travel costs and cost of self-care were directly estimated by each participant, hence not reported here. ^2^ A standardised unit cost was used here to provide a comprehensive and unified basis for costing that reflects a broad spectrum of healthcare services delivered by multidisciplinary team members within the community setting. The reason why the unit cost of GP/nurse/other healthcare practitioners was not individually sourced and applied was because we believe those individually-sourced unit costs were less specific about mental health services provided for older adults. | | | |

**Appendix C. Availability of quality of life and cost data (before imputation)**

| **Type of data** | **Baseline**  **(N=1020), n(%)** | **6-month**  **(N=1020), n(%)** | **Complete case**  **(N=1020), n(%)** |
| --- | --- | --- | --- |
| **Cost data** | | | |
| Resource use from NHS  and PSS perspective | 750 (73.5) | 832 (81.6) | 635 (62.3) |
| Resource use from societal  perspective | 591 (57.9) | 596 (58.4) | 396 (38.8) |
| **Quality of life data** | | | |
| EQ-5D-3L | 1006 (98.6) | 939 (92.1) | 926 (90.8) |
| **Economic evaluation** | | | |
| EQ-5D-3L and costs (NHS  and PSS perspective) | 744 (72.9) | 823 (80.7) | 623 (61.1) |
| EQ-5D-3L and costs  (societal perspective) | 585 (57.4) | 593 (58.1) | 392 (38.4) |
| Abbreviations: NHS: National Health Service; PSS: personal social services | | | |

**Appendix D. Missing pattern of cost and EQ-5D-3L utility**

| 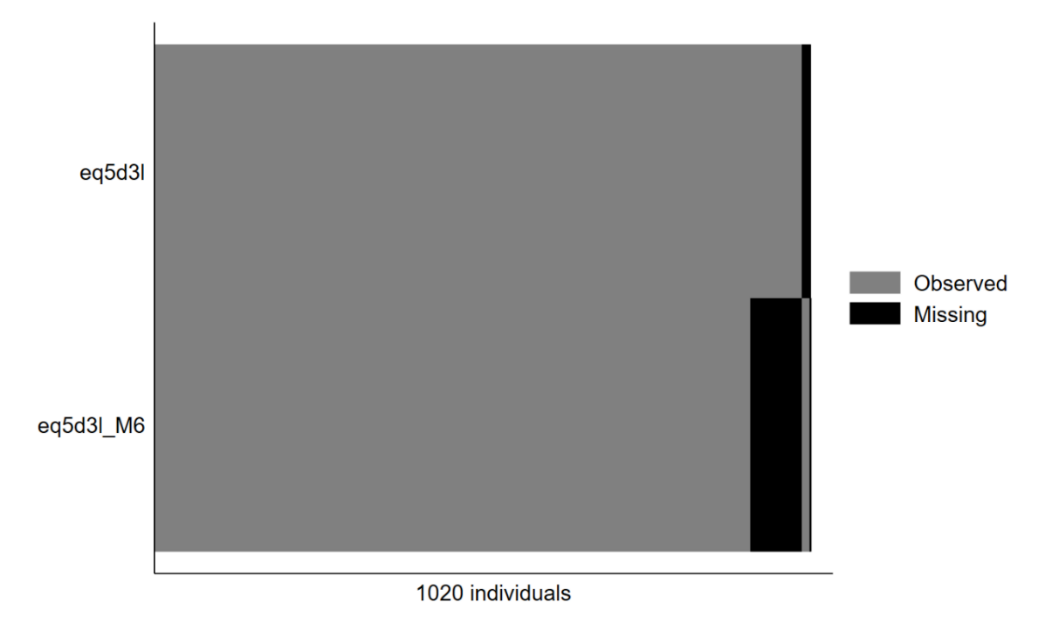  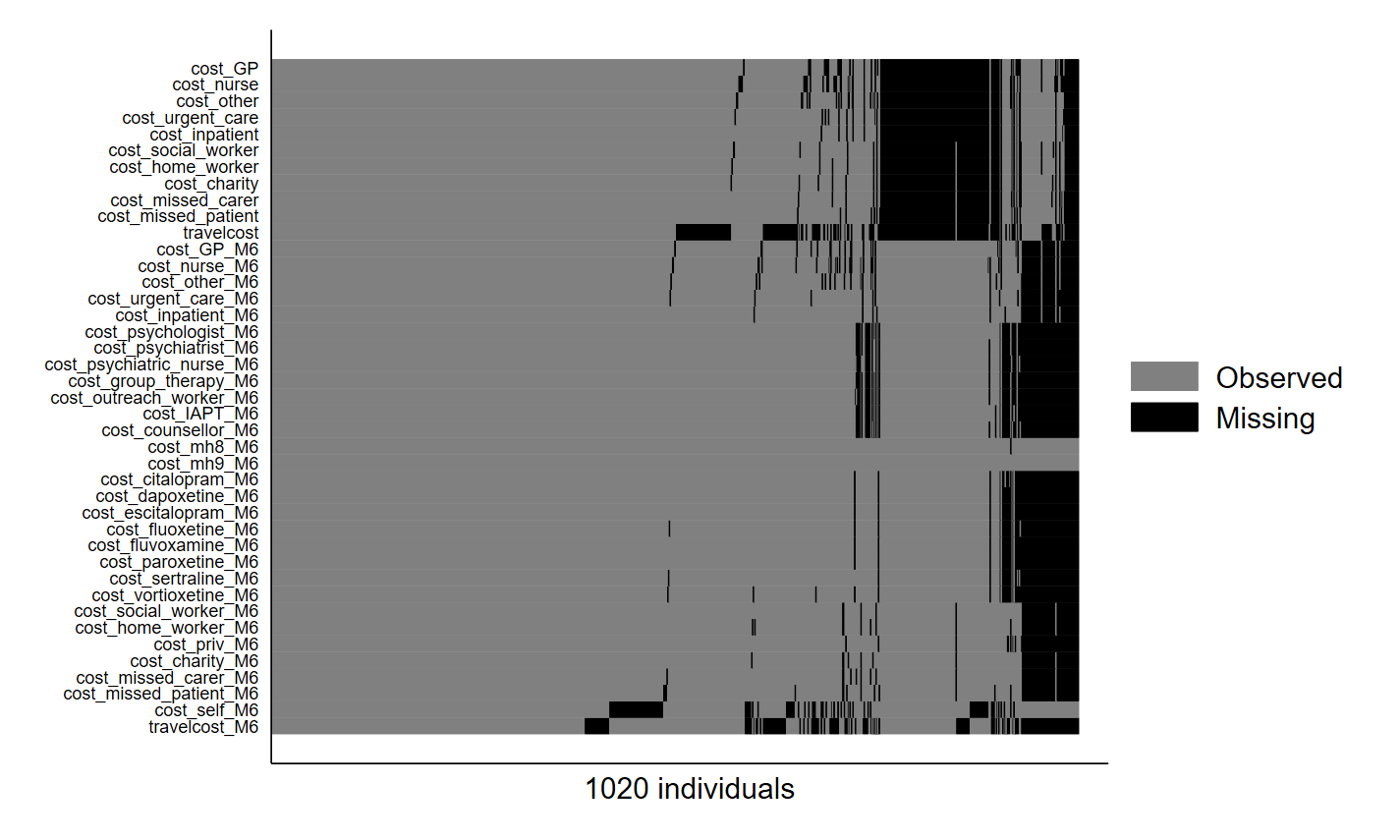 |
| --- |
| According to the missing pattern, the following categories of data were imputed as a whole because they exhibited similar missing pattern, indicating that they could be imputed aggregately:   - cost_community_M0: cost_GP, cost_nurse, cost_other - cost_hospital_M0: cost_urgent_care, cost_inpatient - cost_PSS_M0: cost_social_worker, cost_home_worker - cost_broader_M0: cost_charity, cost_missed_carer, cost_missed_patient - cost_travel_M0: travelcost - cost_community_M6: cost_GP_M6, cost_nurse_M6, cost_other_M6 - cost_hospital_M6: cost_urgent_care_M6, cost_inpatient_M6 - cost_mh_M6: cost_psychologist_M6 - cost_mh9_M6 - cost_med_M6: cost_citalopram_M6 - cost_vortioxetine_M6 - cost_PSS_M6: cost_social_worker_M6, cost_home_worker_M6 - cost_broader_M6: cost_priv_M6, cost_charity_M6, cost_missed_carer_M6, cost_missed_patient_M6 - cost_self_M6: cost_self_M6 - cost_travel_M6: travelcost_M6 |

**Appendix E. Average resource use by treatment group and assessment points: complete case (N=392)**

| Variable | Unit | Time point, mean (SD) | | | |
| --- | --- | --- | --- | --- | --- |
|  |  | Baseline | | 6-month | |
|  |  | SoC  (n=356) | SG  (n=36) | SoC  (n=356) | SG  (n=36) |
| **NHS and PSS** |  |  |  |  |  |
| *Community-based* |  |  |  |  |  |
| GP consultation | session | 0.00 (0.00) | 0.06 (0.23) | 0.00 (0.05) | 0.22 (0.72) |
| Nurse consultation | session | 0.00 (0.00) | 0.00 (0.00) | 0.01 (0.07) | 0.00 (0.00) |
| Other consultation | session | 0.00 (0.00) | 0.03 (0.17) | 0.01 (0.16) | 0.06 (0.23) |
| *Hospital-based* |  |  |  |  |  |
| AE & Urgent Care  Centre | visit | 0.01 (0.26) | 0.00 (0.00) | 0.00 (0.00) | 0.00 (0.00) |
| Inpatient overnight | night | 0.00 (0.00) | 0.00 (0.00) | 0.00 (0.00) | 0.00 (0.00) |
| *PSS-based care* |  |  |  |  |  |
| Social worker | visit | 0.00 (0.00) | 0.00 (0.00) | 0.00 (0.00) | 0.08 (0.50) |
| Home worker | visit | 0.01 (0.07) | 0.25 (1.20) | 0.02 (0.37) | 0.44 (2.35) |
| *NHS mental health services* |  |  |  |  |  |
| Psychologist | session | - | - | 0.00 (0.00) | 0.00 (0.00) |
| Psychiatrist | session | - | - | 0.00 (0.00) | 0.00 (0.00) |
| Psychiatric nurse | session | - | - | 0.00 (0.00) | 0.00 (0.00) |
| Group therapy | session | - | - | 0.00 (0.00) | 0.00 (0.00) |
| Outreach worker | session | - | - | 0.01 (0.17) | 0.03 (0.17) |
| IAPT service | session | - | - | 0.00 (0.00) | 0.00 (0.00) |
| Counsellor | session | - | - | 0.04 (0.64) | 0.00 (0.00) |
| *Medications* |  |  |  |  |  |
| Citalopram | £ | - | - | 0.00 (0.00) | 0.00 (0.00) |
| Dapoxetine | £ | - | - | 0.00 (0.00) | 0.00 (0.00) |
| Escitalopram | £ | - | - | 0.00 (0.00) | 0.00 (0.00) |
| Fluoxetine | £ | - | - | 0.00 (0.00) | 0.00 (0.00) |
| Fluvoxamine | £ | - | - | 0.00 (0.00) | 0.00 (0.00) |
| Paroxetine | £ | - | - | 0.00 (0.00) | 0.00 (0.00) |
| Sertraline | £ | - | - | 0.00 (0.00) | 1.81 (10.83) |
| Vortioxetine | £ | - | - | 0.00 (0.00) | 0.00 (0.00) |
| **Private perspective** |  |  |  |  |  |
| *Charity* | contact | 0.01 (0.17) | 0.14 (0.68) | 0.01 (0.12) | 0.44 (2.67) |
| *Missed work of carers* | day | 0.00 (0.05) | 0.14 (0.59) | 0.00 (0.00) | 0.33 (1.69) |
| *Missed work of patients* | Day | 0.00 (0.00) | 0.00 (0.00) | 0.00 (0.00) | 0.00 (0.00) |
| *Private mental health services* | session | - | - | 0.00 (0.00) | 0.00 (0.00) |
| *Self-care* |  |  |  |  |  |
| Dietary changes | £ | - | - | 2.02 (19.06) | 5.56 (33.33) |
| Exercise | £ | - | - | 14.71 (73.14) | 4.17 (25.00) |
| Herbal, homeopathic  or over the counter  medication | £ | - | - | 1.69 (12.67) | 1.39 (5.93) |
| Hobbies/activities | £ | - | - | 24.54 (104.99) | 27.50 (80.51) |
| Websites or online  resources | £ | - | - | 1.76 (18.41) | 2.22 (13.33) |
| Online forums or  online support groups | £ | - | - | 0.00 (0.00) | 0.00 (0.00) |
| Online videos (e.g.  YouTube) | £ | - | - | 0.20 (2.85) | 0.00 (0.00) |
| Apps | £ | - | - | 0.17 (2.70) | 0.28 (1.67) |
| Podcasts | £ | - | - | 0.00 (0.00) | 0.00 (0.00) |
| Support network  (friends/family) | £ | - | - | 0.14 (2.18) | 1.11 (6.67) |
| Self-help book | £ | - | - | 0.05 (0.90) | 0.03 (0.17) |
| Mindfulness | £ | - | - | 0.00 (0.00) | 0.00 (0.00) |
| Meditation | £ | - | - | 1.46 (26.52) | 16.67 (100.00) |
| Journaling/keeping a  diary | £ | - | - | 0.01 (0.26) | 0.28 (1.67) |
| Abbreviations: SD, standard deviation; SoC, standard of care; SOG, screening + alerting participants and GP. | | | | | |
